# Impact of socio-demographic factors on knowledge, attitude and practices toward scabies among syrian refugees in Jordan: A prospective cross sectional study

**DOI:** 10.1016/j.amsu.2021.102738

**Published:** 2021-08-23

**Authors:** Khaled Seetan, Yasser Rashdan, Adel alsharei, Sharaf al bashir, Abdallah al madani, Mousa alqa'dan, Ashraf al Momani, Hashem al samarah

**Affiliations:** a Department of clinical Sciences, Faculty of Medicine, Yarmouk University, Irbid, Jordan; b Faculty of Medicine, Mutah University, Al Karak, Jordan; c Faculty of Medicine, Jordan University of science and technology, Irbid, Jordan

**Keywords:** Scabies, Knowledge, Refugees, Infestation, Parasites, Pruritus

## Abstract

**Background:**

Scabies is one of the prevalent dermatological conditions, accounting for a substantial proportion of skin diseases in developing countries. It represents a significant health challenge when an outbreak appears in homecare and refugee camps as it may lead to enormous morbidity and high treatment costs. Because Scabies can be easily prevented through education, the purpose of this study was to assess the impact of socio-demographic factors on the level of knowledge, attitude, and practices among Syrian refugees in Jordan's northern region.

**Methods:**

A prospective cross-sectional study was conducted among Syrian refugees attending primary health centers in northern Jordan, Ramtha, Mafraq, and Irbid, from February 2021 to May 2021. The targeted population was adult Syrian refugees above the age of 18. Two thousand participants were included in this study using simple random selection. The study questionnaire included socio-demographic characteristics and knowledge questions such as hearing about Scabies, causes of Scabies, signs and symptoms of Scabies, and its way of transmission. The data was analyzed using (SPSS) version 25.

**Results:**

Females with a mean age of 37.9 years old comprised the majority of the participants. The majority of the participants were married and had intermediate levels of education. The knowledge about Scabies lacked among the majority of the respondents (N = 1259); 321 respondents had moderate knowledge, while only 650 had good knowledge. A statistically significant association was found between knowledge scores and all demographic characteristics, including gender, marital status, income, and educational level.

**Conclusion:**

The general knowledge about Scabies among Syrian refugees is relatively low, with 1259 out of 2000 participants having a bad knowledge score. Moreover, the level of knowledge, attitude, and practices toward Scabies is highly affected by the demographic factors of the Syrian refugees' Health education for refugees is needed to improve their knowledge and help implement prevention programs.

## Highlights

• Scabies is quite a common medical problem among Syrian Refugees in Jordan.
• The socio-demographics of Syrian Refugees affect the level of knowledge and attitude toward scabies infestation.
• The level of knowledge regarding scabies infestation is poor among Syrian Refugees in Jordan.
• Health education for refugees is needed to improve their knowledge and help implement prevention programs.

## 1 Introduction

Scabies is a common skin parasitic infection caused by Sarcoptes scabiei var hominis; it is an endemic disease in tropical and subtropical regions worldwide. It affects more than 130 million individuals worldwide at any given moment. In recent research, rates of scabies incidence range from 0.3% to 46%. In the developed world, outbreaks in health institutions and vulnerable communities contribute to the high economic cost of national health services [[1], [2], [3]]. However, the sheer burden of scabies infestation and complications in resource-poor tropical settings imposes a high cost on healthcare systems. It was predicted in 2010 that direct scabies effects on the skin resulted in more than 1.5 million YLDS (years lived with disability), with the indirect impacts on the renal and cardiovascular function being even more substantial [4].

The 0.4 mm mite makes burrows in the host epidermis to put their eggs. The disease manifestations are mainly due to the infestation by Sarcoptes mites and the host immune response against the parasite's mites, eggs, and other byproducts. The immune response leads to intense itching in response to just a few mites [5]. Scabies infestation is usually complicated by bacterial infection; staphylococci or streptococci are common, leading to the development of skin sores that can cause more severe consequences such as septicemia, heart disease, and chronic kidney disease [[6], [7], [8]].

Scabies is typically transmitted from person to person through physical contact. Transmission through families, mainly from mother to infant, is widespread [9]. Scabies infection is most often a result of unhealthy behavior such as poor personal hygiene, exchanging clothes, and sharing bedding or personal items. Numerous additional elements may contribute to Scabies transmissions, such as high population density, low socioeconomic status, poor environmental conditions, and lack of knowledge about personal hygiene [9].

Schools typically do not provide the level of contact required for transmission. Sexual interaction is frequently the method of transmission among young adults. Nevertheless, we should consider that one single case of Scabies introduced into a crowded community can result in an epidemic [10].

In typical conditions, mites can survive off a host for 24–36 h [11]. Although uncommon, there are numerous documented cases of Scabies contracted by wearing or handling heavily contaminated clothing or sleeping in a bed that an infested individual had previously occupied. Transmission through clothing or linens is more likely with higher parasite burdens, as seen in crusted (Norwegian) Scabies [12,13]. Scabies mites, in general, cannot survive for more than two to three days away from human skin. Appropriate options for items used within several days before treatment (for example, clothing, linens, stuffed animals) include placing them in a plastic bag for at least three days, machine washing with hot water and then ironing or drying in a hot dryer, or dry cleaning [14]. Scabies often occurs among communities with overcrowded living conditions, such as refugee camps, facilitating various contagious diseases, especially skin diseases [15]. Poverty, with its typical consequences; inadequate living conditions, overcrowding, and a low education level seem to be significant driving forces for maintaining a high incidence and prevalence of the disease [16].

Since the Syrian crisis in 2011, many refugees have been displaced to many countries, including Jordan, which has the second-highest share of Syrian refugees, 89 refugees per 1000 inhabitants. 80% of those refugees live below the poverty line, and around 51% were children. Scabies represents a significant health challenge when an outbreak appears in homecare and refugee camps as it may lead to colossal morbidity and high treatment costs. Because Scabies can be easily avoided through education, this study aimed to assess the level of scabies knowledge among Syrian refugees in Jordan's northern region [17].

## 2 Materials and methods

This prospective cross-sectional study was conducted among adult Syrian refugees who attend primary health care centers in northern Jordan (Ramtha, Mafraq, and Irbid) from February 2021 to May 2021. The targeted population was adult Syrian refugees aged 18 years and above who agreed to participate; those who declined to participate in the study were excluded. Two thousand participants were included in this study using simple random selection using the lottery method. Written informed consent was obtained from each of the participants. The IRB committee has approved ethical approval, consent to participate, research protocols, and methods at King Abdullah University Hospital (2712-13-1). All methods were performed by the relevant IRB committee guidelines and regulations. The principles of the World Medical Association and the Declaration of Helsinki were applied in this study. Written informed consent was obtained from all participants in this study. All participants were informed that their comments would be kept anonymous, and no identifying data was collected from them.

The study questionnaire included questions about socio-demographic characteristics of the participants, such as age, gender, level of education, and marital status. The rest of the questionnaire consists of knowledge questions such as hearing about Scabies, causes of Scabies, signs and symptoms of Scabies, and its way of transmission. The data was collected in around four weeks using a structured datasheet. The data was entered into a datasheet, then into an excel document, and analyzed using the statistical package for social science (SPSS) version 25. Categorical variables were described by frequency distribution, while the mean and standard deviation described continuous variables. A Chi-square test was performed for possible associations between the different study variables, a p-value of less than 0.05 was considered statistically significant. This paper was checked to meet all the criteria of STROCSS criteria [25]. This study was registered using researchregistry.com, research ID: researchregistry6991, https://www.researchregistry.com/browse-the registry#home/registration details/60f96bc6c9f208001e405368/.

The level of knowledge was measured using a scoring system with one mark given for each correctly answered question as follows: Good: 10–14, moderate:7–10, poor: less than 7.

## 3 Results

Females with a mean age of 37.99 years old comprised the majority of the participants. Most of the participants were married and had intermediate levels of education (Table 1).

**Table 1:** Demographic characteristics of the Syrian refugees in northern Jordan (n = 2230).

	Frequency/mean ± SD	Percent
Age	37.99 ± 13.2	
Gender		
Male	1085	48.7
Female	1145	51.3
Marital status		
Single	543	24.3
Married	1638	73.5
Divorced	22	1
Widowed	27	1.2
Income ($)		
Less than 250	730	32.7
Between 250 and 500	1164	52.2
More than 500	336	15.1
Educational level		
Primary	282	12.6
Intermediate	1634	73.3
Secondary	19	0.9
Bachelor	295	13.2

**Fig. 1:**
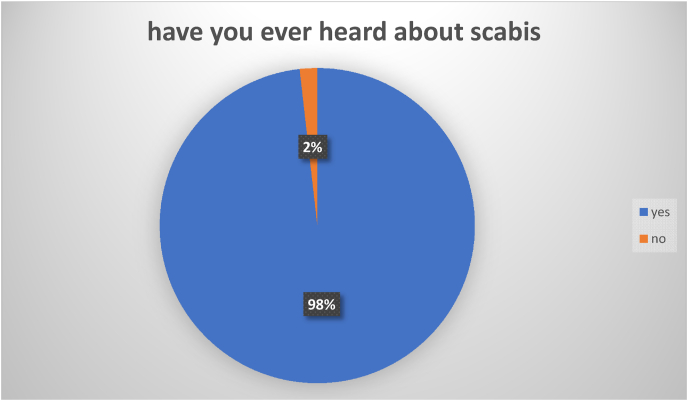
Proportion of participants heard about Scabies among Syrian refugees in northern Jordan (n = 2230).

**Fig. 2:**
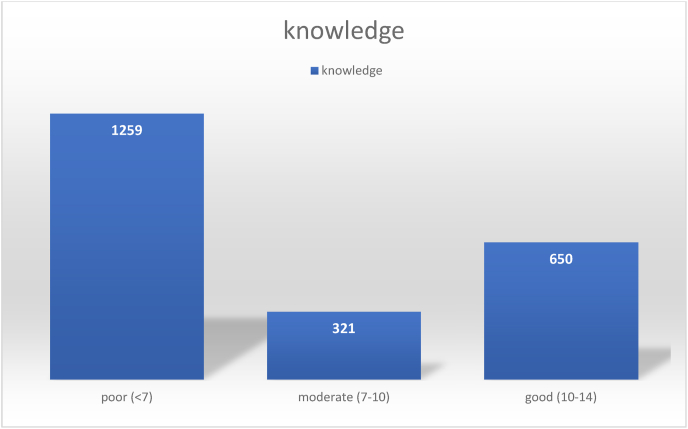
Illustration of Syrian refugees' knowledge about Scabies, northern Jordan (n = 2000).

Although only 2% of the participants had not ever heard of Scabies (Fig. 1), the level of knowledge about Scabies was poor (less than 7) among the majority of the respondents (N = 1259); 321 had moderate knowledge (7–10) while only 650 had good knowledge (10–14) about Scabies (Fig. 2).

In general, 69.7% of the respondents agreed that a skin parasite causes Scabies; however, most of the participants, 84.4%, reported that scabies cause no itching. The level of knowledge about scabies transmission mode was poor as nearly one-third of the respondents believed that Scabies could be transmitted by direct body contact, while about half of the respondents stated that they do not know if Scabies can be transmitted by blood and droplets or not, moreover, 47% agreed that Scabies could be transmitted through sharing of clothes and towels.

About 48.3% of the participants agreed that Scabies is not self-limiting and that infected patients should be isolated. Only one-third of the participants agreed that taking care of personal hygiene helps prevent Scabies transmission. The majority believed that treatment should start immediately following the diagnosis and all family members of the infected patients need to be examined and followed. Nearly half of the participants agreed that there is no need for environmental disinfection by pesticides following the diagnosis. The majority of participants agreed that Scabies could be very harmful and severe; they also did not know if Scabies only affects older people or not, and they agreed that daily body washing helps clear Scabies (Table 2).

**Table 2:** Syrian refugees’ knowledge about Scabies, Northern Jordan (n = 2230).

	Count.	%
Scabies is caused by a skin parasite		
Yes*	1555	69.7
No	355	15.9
I do not know	320	14.3
Scabies causes no itching.		
Yes	1883	84.4
No*	337	15.1
I don't know	10	0.4
Direct body contact can transmit scabies.		
Yes*	845	37.9
No	1025	46
I don't know	360	16.1
Scabies can be transmitted through blood and droplets		
Yes	57	2.6
No*	1048	47
I don't know	1125	50.4
Scabies is not transmitted through sharing clothes and towel of an infested patient.		
Yes	271	12.2
No*	1049	47
I don't know	910	40.8
There is no need to isolate infested patients		
Yes	751	33.7
No*	921	41.3
I don't know	558	25
There is no need to isolate infested patients as it is self-limiting		
Yes	701	31.4
No*	1076	48.3
I don't know	453	20.3
Taking care of personal hygiene helps to prevent scabies transmission		
Yes*	709	31.8
No	1329	59.6
I don't know	192	8.6
All family members of infested patients need to be examined and followed.		
Yes*	1637	73.4
No	142	6.4
I don't know	451	20.2
Treatment should start immediately following diagnosis.		
Yes*	1531	68.7
No	378	17
I don't know	321	14.4
Environmental disinfestation using pesticide spray is essential following diagnosis.		
Yes	530	23.8
No*	1287	57.7
I don't know	413	18.5
Scabies affects only old age people.		
Yes	428	19.2
No*	650	29.1
I don't know	1152	51.7
Scabies can be very harmful and serious		
Yes	1508	67.6
No*	452	20.3
I don't know	270	21.1
Daily body wash helps clear scabies.		
Yes	1656	74.3
No*	265	11.9
I don't know	309	13.9

The study showed a strong inverse correlation (Pearson correlation -.811, P-value 0.000) between the age of the participants and Scabies knowledge score (Table 3). There was also a statistically significant association between knowledge score and all demographic characteristics, including gender, marital status, income, and educational level, P-value <0.001 (Table 4).

**Table 3:** Correlation between age and scabies knowledge scores among Syrian refugees in Northern Jordan (n = 2230).

		Knowledge score
age	Pearson Correlation	-.811**
	p-value	.000
	N	2230

**Table 4:** Comparison of knowledge scores across demographics of the Syrian refugees in Northern Jordan (n = 2230).

	Mean ± SD	p-value
Gender		<0.001
Male	7.9 ± 3	
Female	4.1 ± 2	
Marital status		<0.001
Single	8.3 ± 1	
Married	5.3 ± 3.3	
Divorced	3.6 ± 0.5	
Widowed	2.3 ± 0.5	
Income		<0.001
Less than 250	3.8 ± 2.1	
Between 250 and 500	6.3 ± 3.1	
More than 500	9.6 ± 2	
Educational level		<0.001
Primary	2.3 ± 0.5	
Intermediate	5.7 ± 3	
Secondary	11	
Bachelor	10.5 ± 0.5	

The difference between the marital status groups was significant except between widowed and divorced (Table 5). The difference in the knowledge score is significantly different among the three different income groups (Table 6). A significant difference was found between the education levels and the knowledge score, except for the difference between secondary and bachelor's degrees (Table 7).

**Table 5:** Post-hoc test for mean differences of knowledge scores among marital status groups of the Syrian refugees in Northern Jordan (n = 2230).

Tukey HSD						
(I) income	(J) income	Mean Difference (I-J)	Std. Error	Sig.	95% Confidence Interval	
					Lower Bound	Upper Bound
Less than 250	Between 250 and 500	−2.51597^a^	.12731	.000	−2.8145	−2.2174
	More than 500	−5.85887^a^	.17777	.000	−6.2758	−5.4419
Between 250 and 500	Less than 250	2.51597^a^	.12731	.000	2.2174	2.8145
	More than 500	−3.34291^a^	.16700	.000	−3.7346	−2.9512
More than 500	Less than 250	5.85887^a^	.17777	.000	5.4419	6.2758
	Between 250 and 500	3.34291^a^	.16700	.000	2.9512	3.7346

a The mean difference is significant at the 0.05 level.

**Table 6:** Post-hoc test for mean differences of knowledge scores among income groups in the Syrian refugees in Northern Jordan (n = 2230).

Tukey HSD						
(I) marital status	(J) marital status	Mean Difference (I-J)	Std. Error	Sig.	95% Confidence Interval	
					Lower Bound	Upper Bound
Single	Married	3.09124^a^	.14822	.000	2.7102	3.4723
	Divorced	4.74669^a^	.65096	.000	3.0731	6.4203
	Widowed	6.08676^a^	.59019	.000	4.5694	7.6041
Married	Single	−3.09124^a^	.14822	.000	−3.4723	−2.7102
	Divorced	1.65546^a^	.64243	.049	.0038	3.3071
	Widowed	2.99552^a^	.58077	.000	1.5024	4.4887
Divorced	Single	−4.74669^a^	.65096	.000	−6.4203	−3.0731
	Married	−1.65546^a^	.64243	.049	−3.3071	-.0038
	Widowed	1.34007	.85969	.403	-.8702	3.5503
Widowed	Single	−6.08676^a^	.59019	.000	−7.6041	−4.5694
	Married	−2.99552^a^	.58077	.000	−4.4887	−1.5024
	Divorced	−1.34007	.85969	.403	−3.5503	.8702

a The mean difference is significant at the 0.05 level.

**Table 7:** Post-hoc test for mean differences of knowledge scores among educational level groups of the Syrian refugees in Northern Jordan (n = 2230).

Tukey HSD						
(I) educational level	(J) educational level	Mean Difference (I-J)	Std. Error	Sig.	95% Confidence Interval	
					Lower Bound	Upper Bound
Primary	Intermediate	−3.36256^a^	.16293	.000	−3.7814	−2.9437
	Secondary	−8.61348^a^	.59887	.000	−10.1532	−7.0738
	Bachelor	−8.07449^a^	.21043	.000	−8.6155	−7.5335
Intermediate	Primary	3.36256^a^	.16293	.000	2.9437	3.7814
	Secondary	−5.25092^a^	.58303	.000	−6.7499	−3.7520
	Bachelor	−4.71193^a^	.15984	.000	−5.1229	−4.3010
Secondary	Primary	8.61348^a^	.59887	.000	7.0738	10.1532
	Intermediate	5.25092^a^	.58303	.000	3.7520	6.7499
	Bachelor	.53898	.59804	.804	-.9986	2.0765
Bachelor	Primary	8.07449^a^	.21043	.000	7.5335	8.6155
	Intermediate	4.71193^a^	.15984	.000	4.3010	5.1229
	Secondary	-.53898	.59804	.804	−2.0765	.9986

a The mean difference is significant at the 0.05 level.

## 4 Discussion

This analytical cross-sectional study was conducted to evaluate the knowledge of the Syrian refugees in the northern area of Jordan about Scabies, it's transmission, and prevention. It was investigating the knowledge about the case of the disease, its signs and symptoms, ways of transmission, whether it is self-limiting or not, the importance of early treatment, and the seriousness of the disease. The study also investigates the association between the demographic and the knowledge score.

This study finds an overall poor level of knowledge about Scabies among the study participants, In contrast to previous studies done in Saudi Arabia 2018 [18], and Indonesia where a good level of knowledge was found among the majority of the participants [4].

While these findings are comparable to another study done among medical students living in an endemic region, overall unsatisfactory knowledge of Scabies has been reported [19].

In general, 69.7% of the respondents agreed that a skin parasite causes Scabies; however, the majority of the participants, 84.4%, showed agreement with the statement that scabies cause no itching. Considering that Pruritus, the result of a hypersensitive reaction to components of the saliva, eggs, and other material of the mites, is the main symptom of the disease, it typically worsens at night and can prevent patients from sleeping well [20].

These study findings contrast with what has been found in another previous study, where 93.3% of the participants knew that scratching is a prominent symptom of Scabies [21]. Only 31.8% of the study participants agreed that taking care of personal hygiene helps prevent Scabies transmission. The spread of classic Scabies without direct person-to-person contact is rare. However, the recovery of mites from chairs and beds in patients' homes with Scabies supports the role of personal hygiene and other environmental measures [22]. Even though data are lacking to confirm the efficacy of some measures in reducing transmission, ideally, Clothing and bedding should be washed at 60 °C and dried the day after the first treatment [23].

The majority agrees that treatment should begin as soon as the diagnosis is made; these findings are consistent with those found in a previous study conducted in Guinea-Bissau, where participants recognized the importance of early treatment but were unaware that personal hygiene is essential for Scabies prevention and recurrence [24].

In order to have reasonable control over Scabies infection, treatment of affected individuals, as well as their contacts, is of vast importance [20]. Prescriptions should be provided for all household members and any sexual contacts, even if they are asymptomatic [12].

Treatment of close relatives was considered unnecessary by previous study participants, while in this study, the majority agreed that all family members of the infected patients needed to be examined and followed [24].

A statistically significant association was found between the knowledge score and all demographic characteristics, including age, gender, marital status, income, and educational level, which help explain the difference in the knowledge found between this study and the previous studies [4,18,21,24]. Furthermore, it also goes with a previous study where the results suggest a statistically significant association between marital status and level of knowledge about Scabies [19].

The major limitation that faced the study was the outbreak of the COVID-19, it had limited us to the primary health care centers, and it had also limited the patients' attendance to primary health care centers. We suggest further explorative researches be done at the level of communities, not facility-based researches.

### 4.1 Limitations

Although more than two thousand adults responded to this prospective cross-sectional study, their opinions are self-reflected and may not reflect the behaviors of the entire population of the study. As this is a cross-sectional study, the temporal relation between the insufficient knowledge about Scabies, the variable demographic factors, and the high prevalence of Scabies among the targeted population cannot be determined.

## 5 Conclusion

The general knowledge about Scabies among Syrian refugees is relatively low, with 1259 out of 2000 participants having a bad knowledge score. Moreover, the level of knowledge, attitude, and practices toward Scabies is highly affected by the demographic factors of the Syrian refugees. Health education for refugees is needed to improve their knowledge and help implement prevention programs.

## Ethical approval

Ethical approval is received according to the ongoing regulations of King Abdullah University Hospital.

## Source of funding

The authors declared that this study had received no financial support.

## Author contribution

All authors read and approved the content of the submitted study.

## Registration of research studies

Not Applicable.

## Guarantor

Khaled Seetan.

## Research consent

Consent was obtained from all the volunteers.

## Declaration of competing interest

The authors report no conflict of interest.

## Provenance and peer review

Not commissioned, externally peer-reviewed.
